# Editorial: Harnessing the power of small RNAs: advances and applications in plant gene silencing and stress response

**DOI:** 10.3389/fpls.2026.1924418

**Published:** 2026-07-10

**Authors:** Humberto Prieto, Álvaro Castro

**Affiliations:** 1 Laboratorio de Biotecnología, Instituto de Investigaciones Agropecuarias, La Platina Research Station, Santiago, Chile; 2 University of California (UC) Davis Chile Life Sciences Innovation Center, Santiago, Chile

**Keywords:** RNA interference (RNAi), small RNAs (sRNAs), spray induced gene silencing (SIGS), stress response, systemic RNA mobility

## Introduction

Small RNAs (sRNAs) have emerged as central regulators of plant gene expression, orchestrating processes that span development, stress adaptation, genome stability and cross kingdom communication. The contributions gathered in this Research Topic illustrate the breadth and depth of contemporary small RNA biology, ranging from mechanistic insights into biogenesis and trafficking to applied innovations in RNA guided crop improvement. Collectively, these studies highlight how miRNAs, siRNAs and their artificial derivatives, including artificial microRNAs (amiRNAs) and artificial small interfering RNAs (asiRNAs), constitute a multilayered regulatory network that plants deploy to sense, interpret and respond to their environment.

A recurring theme across the contributions is the dynamic interplay between endogenous sRNA pathways and plant stress responses. Singh et al. provide a compelling example of cross kingdom RNA trafficking in the Phytophthora infestans and potato interaction, where a pathogen derived small RNA is predicted to target the host immune regulator EDS1. Their work also reveals the selective enrichment of pathogen RNAs in extracellular vesicles, offering mechanistic evidence for vesicle mediated RNA exchange. This aligns with the conceptual framework presented by De Meo et al., who review how RNA interference shapes Arabidopsis stress responses through AGO specialization and systemic sRNA mobility. This mobility supports both post transcriptional gene silencing and transcriptional gene silencing through the RNA directed DNA methylation pathway, a mechanism essential for long term genomic stability and adaptation.

Regulatory versatility is further expanded by technological advances in nucleotide level detection. Xiang et al. provide a comprehensive review of the m6A epitranscriptomic landscape and its intersection with sRNA pathways. They highlight methodological milestones, including scm6A seq and GLORI, that enable single nucleotide resolution and absolute quantification, transforming our capacity to map dynamic RNA modifications under environmental cues. This epitranscriptomic dimension resonates with the analysis by Lin et al., who examine mobile RNAs as systemic signals that coordinate whole plant responses to nutrient deficiency and parasitic interactions. Their synthesis reveals that mobile miRNAs, siRNAs and lncRNAs form long distance communication networks that integrate environmental perception with developmental reprogramming.

At the cellular level, Bian et al. employ single cell RNA sequencing to dissect transcriptional heterogeneity in maize roots. By identifying cell type specific expression patterns of hormone related genes, their work provides a high resolution framework for understanding how sRNA mediated regulation varies across cell identities. Such spatial resolution is essential for interpreting the cell specific action of miRNAs and secondary siRNAs in tissues where developmental and stress pathways intersect.

The functional consequences of sRNA mediated regulation are exemplified by Wang et al., who characterize the EIN3 and EIL transcription factor family in rye. Their findings align with the broader theme of regulatory crosstalk, since EIN3 and EIL proteins act as integration nodes where ethylene signaling converges with miRNA regulated developmental and defense pathways. This reinforces the idea that sRNA mediated regulation is deeply embedded within hormonal and stress responsive networks that shape plant resilience under fluctuating environmental conditions.

Beyond endogenous pathways, several contributions address the biosafety and applied potential of artificial RNAi technologies. Li et al. evaluate the ecological safety of dsRNA expressing cotton and report minimal off target effects on the beneficial predator Orius similis. Their work establishes a rigorous framework for assessing unintended impacts, an essential step for developing regulatory standards and public acceptance as RNAi based crops move toward broader agricultural deployment.

A key contribution that completes this Research Topic is the study by Xu et al., who present the first comprehensive genome wide analysis of the DCL, AGO and RDR gene families in autotetraploid alfalfa. Their work identifies extensive gene family expansion driven by segmental duplication, promoter regions enriched in stress responsive cis elements and tissue specific expression patterns. Importantly, they validate core stress responsive candidates under salt and drought conditions, providing a foundational resource for understanding how RNA silencing machinery contributes to abiotic stress resilience. This study bridges mechanistic RNA biology with practical breeding applications and strengthens the connection between small RNA pathways and crop improvement.

## Looking forward: exogenous RNA technologies and the next frontier

**Figure 1:**
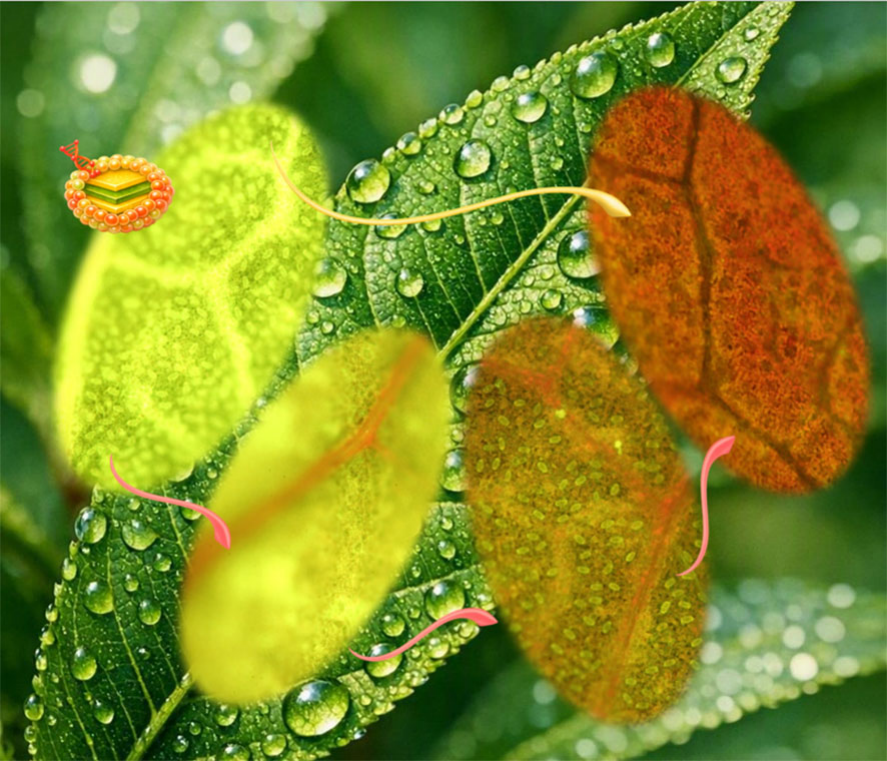
Progressive GFP silencing in adult transgenic peach leaves following topical application of artificial miRNA delivered via LDH–siRNA complexes. Representative leaves from mature GFP-expressing Prunus persica trees were treated with LDH-adsorbed artificial miRNA targeting GFP. The color transition sequence (from an initial green state to vein−localized reddening, followed by expansion across the lamina and culminating in near−complete silencing of the leaf blade) illustrates the gradual reduction of fluorescence and chlorophyll emission associated with silencing progression. The LDH–siRNA nanocomplex depicted (upper left) corresponds to the particles formulated and supplied by ApoloBiotech (Argentina) for these assays. These experiments, generated in our group at the Biotechnology Laboratory, INIA−Chile, illustrate preliminary observations consistent with the potential of exogenous RNA−based silencing in adult woody tissues.

The convergence of mechanistic insight, high resolution profiling and applied innovation points toward a rapidly evolving future for RNA based plant biotechnology. Spray Induced Gene Silencing represents a particularly promising non transgenic platform for precision crop protection (**Figure 1**). However, its success depends on overcoming challenges such as RNA instability, environmental degradation and limited uptake efficiency. Advances in nanocarrier mediated delivery, including layered double hydroxides, liposomes and polymeric nanoparticles, are beginning to address these limitations by protecting sRNAs from nucleases and facilitating vascular transport.

Mechanistic findings on vesicle mediated RNA trafficking, together with principles of systemic sRNA mobility, suggest that plants already possess sophisticated endogenous pathways for RNA movement. Harnessing or mimicking these pathways, potentially through engineered vesicles, stabilized dsRNA formulations or targeted delivery systems, may define the next generation of SIGS and RNAi technologies. As these innovations mature, they hold the potential to deliver highly specific and environmentally sustainable solutions for crop protection and improvement.

Together, the contributions in this Research Topic illuminate the expanding landscape of small RNA biology and its translational potential. By bridging mechanistic understanding with technological innovation, they showcase the transformative power of sRNAs as biological regulators and biotechnological tools, positioning RNA guided strategies at the forefront of plant science and future agriculture.

This editorial was written by Humberto Prieto and Álvaro Castro, acting on behalf of the full editorial team. The authors developed the conceptual synthesis and thematic integration of the text, reflecting the collective vision of the four editors who contributed to the curation and overall review of this Research Topic.

